# c-Jun N-terminal kinase 1 defective CD4+CD25+FoxP3+ cells prolong islet allograft survival in diabetic mice

**DOI:** 10.1038/s41598-018-21477-9

**Published:** 2018-02-19

**Authors:** Deepak Tripathi, Satyanarayana S. Cheekatla, Padmaja Paidipally, Rajesh Kumar Radhakrishnan, Elwyn Welch, Ramya Sivangala Thandi, Amy R. Tvinnereim, Ramakrishna Vankayalapati

**Affiliations:** 1 Department of Pulmonary Immunology, Center for Biomedical Research, University of Texas Health Science Center at Tyler, Tyler, Texas 75708 USA; 2 Present Address: Department of Biotechnology, Gandhi Institute of Technology and Management (GITAM) Institute of Science, GITAM University, Visakhapatnam, Andhra Pradesh 530045 India

## Abstract

CD4+CD25+FoxP3+ cells (Tregs) inhibit inflammatory immune responses to allografts. Here, we found that co-transplantation of allogeneic pancreatic islets with Tregs that are defective in c-Jun N-terminal kinase 1 (JNK1) signaling prolongs islet allograft survival in the liver parenchyma of chemically induced diabetic mice (CDM). Adoptively transferred JNK1^−/−^ but not wild-type (WT) Tregs survive longer in the liver parenchyma of CDM. JNK1^−/−^ Tregs are resistant to apoptosis and express anti-apoptotic molecules. JNK1^−/−^ Tregs express higher levels of lymphocyte activation gene-3 molecule (LAG-3) on their surface and produce higher amounts of the anti-inflammatory cytokine interleukin (IL)-10 compared with WT Tregs. JNK1^−/−^ Tregs inhibit liver alloimmune responses more efficiently than WT Tregs. JNK1^−/−^ but not WT Tregs are able to inhibit IL-17 and IL-21 production through enhanced LAG-3 expression and IL-10 production. Our study identifies a novel role of JNK1 signaling in Tregs that enhances islet allograft survival in the liver parenchyma of CDM.

## Introduction

CD4+CD25+FoxP3+ cells (Tregs) constitute 5–10% of CD4+ T cells in mice and humans and are essential for the maintenance of peripheral tolerance and homeostasis^1^. These cells express CD25 on the cell surface and a transcriptional regulator, Foxp3. The development and function of Tregs depends on Foxp3 expression^1^. Most Tregs arise during normal maturation in the thymus and survive in the periphery as “natural T regs”^2^. Some Tregs develop from conventional CD4+ T cells in response to infectious challenge and are called inducible Tregs or antigen-specific Tregs^3^. Tregs are anergic, require interleukin (IL)-2 for survival and growth, and suppress proliferation and functional activity of CD4+CD25− cells^4^. Several studies have demonstrated that Tregs can prevent autoimmunity, suppress the immune response to tumors, and inhibit transplant graft rejection^1,5^.

In various transplant models, it has been shown that Tregs inhibit inflammatory immune responses to allografts^6^. Despite extensive studies on the role of Tregs in allograft survival models, adoptively transferred Tregs alone remain ineffective in the induction of long-term allograft survival^7^, mainly because Tregs are unable to survive, expand and exhibit immunosuppressive function long term^8^. It has also been shown that Tregs are unstable and dysfunctional in the presence of inflammatory cytokines^9^. Tregs express transcription factors such as retinoid-related orphan receptor γ (*RORγ*), and inflammatory cytokines can inhibit their immunosuppressive capacity and induce Th17 cytokine production^10–12^. This finding suggests that it is important to modify Tregs to increase their survival and immunosuppressive potential before using them for immunosuppressive therapies. Like other effector T-cells, Tregs express several signaling molecules^13^. Among these signaling molecules, c-Jun N-terminal kinases (JNKs) are a group of protein kinases that regulate diverse cellular functions including T cell growth, differentiation and apoptosis^14–17^. Previous studies have demonstrated that the absence of c-Jun N-terminal kinase (JNK) 1 promotes IL-4, IL-10 and transforming growth factor (TGF)-β production by T cells^14,18^.

In the current study, using an islet allograft transplantation model, we determined that JNK1-defective Tregs prolong islet allograft survival in liver parenchyma more efficiently than wild-type (WT) Tregs. The immunosuppressive environment in the kidney capsule, testis, and liver can protect allogeneic islets from inflammation-mediated destruction in comparison to other organs^19–21^. It has been demonstrated that islet allografts can survive long term in the liver parenchyma^21,22^. We transplanted an islet allograft into the hepatic parenchyma of chemically induced diabetic (CDM) or non-obese diabetic (NOD) recipient mice with or without JNK1-defective or WT Tregs and determined the islet allograft survival. We found that JNK1-defective Tregs are less apoptotic, survive for a longer period of time and show prolonged islet allograft survival compared with WT Tregs. We also found that JNK1-defective Tregs inhibit IL-17 and IL-21-mediated inflammation through the production of IL-10 and expression of LAG (lymphocyte activation gene) -3.

## Results

### JNK1^−^defective Tregs prolong islet allograft survival in liver parenchyma of CDM

**Figure 1:**
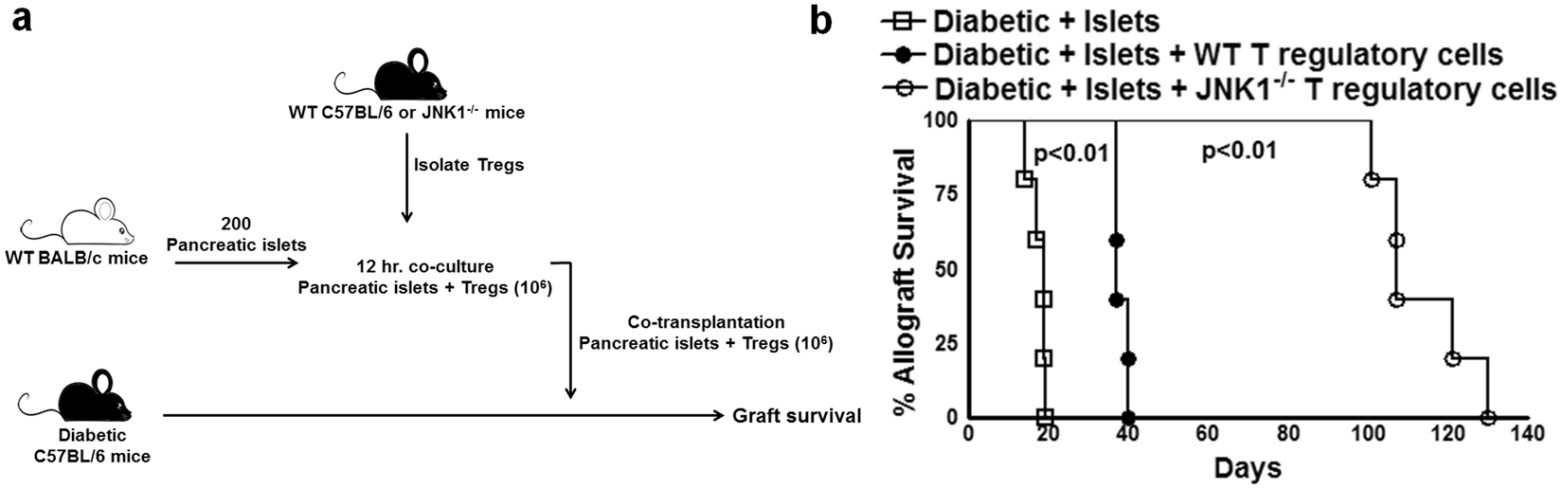
JNK1 defective Tregs prolong islet allograft survival in liver parenchyma of CDM. A single intraperitoneal injection of streptozotocin (STZ) (180 mg/kg body weight) caused C57BL/6 mice to develop diabetes, as measured by random blood sugar levels after one week. Approximately 200 pancreatic islets obtained from BALB/c mice (donor) were cultured in medium for 12 hours and transplanted into the liver parenchyma of CDM (recipient). Some of the islet allograft recipient mice received CD4+CD25+Foxp3+ cells (10^6^) from WT or JNK^−/−^ mice (both C57BL/6 background) that were isolated and cultured with islets for 12 hours prior to transfer along with islets as mentioned in the Methods section. Blood glucose levels were measured every 72 hours up to 140 days (**a**). Schematic representation of CD4+CD25+Foxp3+ cell-loaded islets. Blood glucose levels above 300 mg/deciliter were considered to indicate diabetes or failed glucose control (**b**). Percent allograft survival. The percent graft survival was calculated using the log-rank test. Kaplan–Meier survival curves of the mice are shown. Data presented are representative of five independent experiments. Five mice per group were used.

We determined the role of CD4+CD25+FoxP3+ cells (Tregs) in allograft tolerance in a pancreatic islet transplantation model. First, we chemically induced diabetes in C57BL/6 mice as described in the Methods section. Next, we isolated pancreatic islets from control BALB/c mice, cultured them for 12 hours, and then transplanted 200 islets into the liver parenchyma of the above-mentioned C57BL/6 CDM. Some of CDM received 200 islets loaded with 10^6^ Tregs (cultured with islets for 12 hours before transplantation) isolated from spleens of control non-diabetic C57BL/6 mice (Fig. 1a). We measured non-fasting blood glucose every third day and considered a graft rejected if the glucose levels exceeded 300 mg/dl (Supplementary Fig. 1a). The allogeneic islet grafts survived up to 18 days in the liver parenchyma of recipient mice without any treatment (Fig. 1b). The islet allograft survived up to 40 days when co-cultured and transplanted together with Tregs (Fig. 1b, p < 0.01). We also determined the survival of allogeneic pancreatic islets in the liver parenchyma of the NOD mice. We isolated pancreatic islets from C3H/HeJ (MHC mismatch to NOD mice) and Tregs from 8-week-old NOD mice after 12 h co-culture. The islets were transplanted into the thirteen-week-old hypoglycemic NOD mice liver parenchyma. Some NOD recipient mice were treated with low doses of rapamycin of 0.2 or 0.4 mg/kg/daily for 14 days. The allogeneic islets grafts in the NOD mouse liver parenchyma survived up to 10 days, and further survival was enhanced up to 35 days when co-transplanted with Tregs (Supplementary Fig. 1c,d). Treg-co-transplanted NOD mice gained weight 10 days post-transplant and maintained their weight until termination of the experiment (Supplementary Fig. 1e).

JNK1 signaling regulates the expression of multiple transcriptional factors and induces the apoptosis of T-cells^15,23^. JNK1 signaling also inhibits the suppressive function of Tregs^24,25^. We asked whether JNK1-defective Tregs further prolong islet survival. In the above experiment, some of the C57BL/6 CDM received Tregs from JNK1^−/−^ mice instead of Tregs from WT mice along with the BALB/c mice pancreatic islets. JNK1^−/−^ Tregs prolonged islet allograft survival up to 120 days (Fig. 1b, p < 0.01). CDM that received WT mouse Tregs along with allogeneic islets initially gained weight compared with CDM that did not receive the islets and lost weight later when the islet grafts were rejected. Similarly, CDM that received JNK1^−/−^ mouse Tregs along with allogeneic islets gained weight and maintained the same weight until 85 days after transplantation and further gained more weight (Supplementary Fig. 1b). These results demonstrate that JNK1^−/−^ Tregs are more efficient at prolonging islet allograft survival in the liver parenchyma.

### Lack of JNK1 expression enhances the survival of Tregs in recipient mouse liver parenchyma

**Figure 2:**
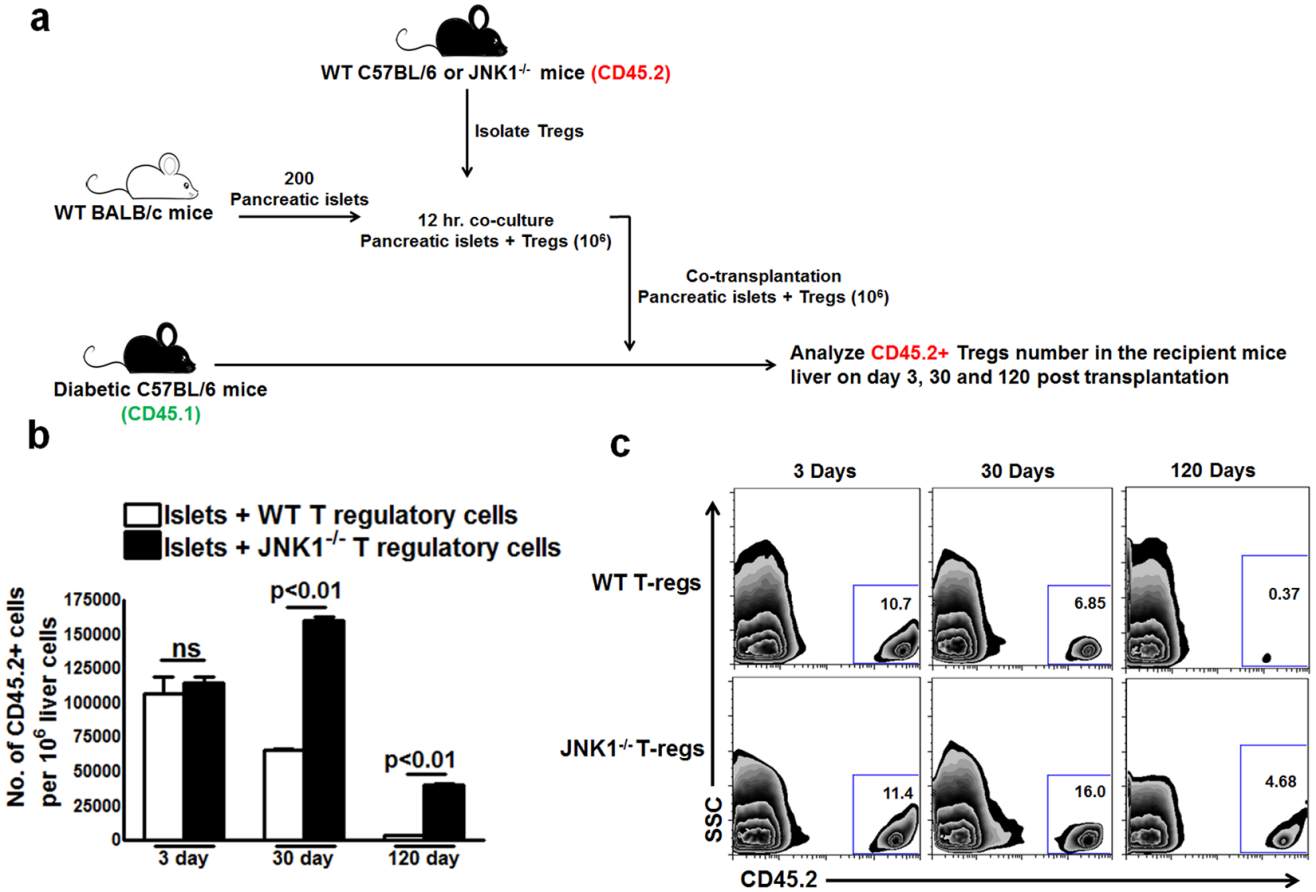
Lack of JNK1 expression enhances survival of Tregs in recipient mice. Pancreatic islets and Tregs from WT or JNK1^−/−^ mice (CD45.2) were transferred to diabetic mice (CD45.1) as in Fig. 1a. Three, thirty and one hundred twenty days after transplantation, the percentages of donor-derived (CD45.2) cells in recipient mouse liver were determined by flow cytometry. **(a)** Schematic representation of the experiment. (**b**) Absolute number of cells. (**c**) A representative flow cytometry figure. Data presented are representative of five independent experiments. Five mice per group were used. Mean values, p values and SEs are shown.

We asked whether increased islet graft survival in the liver parenchyma is due to increased survival of JNK1^−/−^ Tregs in the liver parenchyma. BALB/c mouse islets were transplanted into liver parenchyma of CDM (CD45.1 background) along with WT or JNK1^−/−^ CD4+CD25+ Tregs (CD45.2 background), as mentioned in the Methods section and in Fig. 2a. The number of donor-derived Tregs (CD45.2) in the liver of CDM recipients (CD45.1) was determined by flow cytometry after 3, 30 and 120 days of transplantation. As shown in Fig. 2b, three days after transplantation, the number of donor-derived cells (CD45.2+ WT and JNK1^−/−^ Tregs) were similar in the liver of CDM recipients (CD45.1). Thirty days after transplantation, the number of CD45.2+ WT Tregs (65475.0±1865.0) were significantly less than the number of CD45.2+ JNK1^−/−^ Tregs (160245.0 ± 5320.0) in the liver parenchyma of CDM recipient mice (CD45.1). After 120 days, the number of CD45.2+ WT Tregs was 11-fold less than CD45.2+ JNK1^−/−^ Tregs (3700.0 ± 258.0 *vs*. 40250.0 ± 2045) in the liver parenchyma of CD45.1+ recipient mice (Fig. 2b and Supplementary Fig. 1f).

### JNK1^−/−^ Tregs are less apoptotic and express higher levels of anti-apoptotic genes myeloid leukemia cell differentiation protein-1 (Mcl-1) and B-cell lymphoma-extra-large (Bcl-xL) than WT Tregs

**Figure 3:**
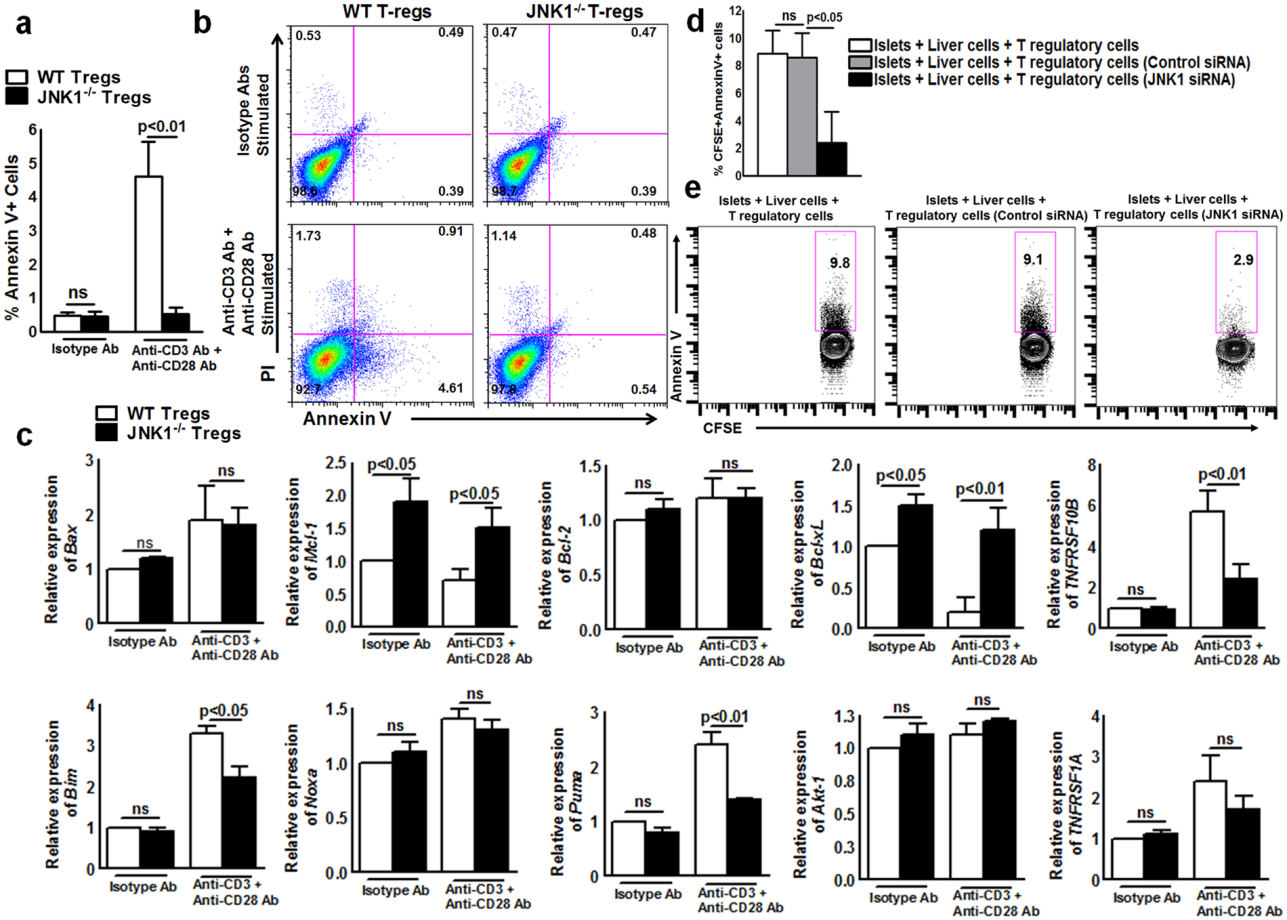
JNK1^−/−^ Tregs are less apoptotic than WT Tregs. a to c. CD4+CD25+ Tregs from WT or JNK1^−/−^ were stimulated with isotype control antibodies IgG and IgG2 or anti-CD3 (5 μg/ml) and anti-CD28 (1 μg/ml) as described in the Methods section. After six hours, (**a**) the percentage of Annexin V+ cells was determined by flow cytometry. **(b)** A representative flow cytometry figure. (**c**) The mRNA expression of pro- and anti-apoptotic genes (*Bax, Mcl-1, Bcl-2, Bcl-Xl, TNFRSF10B, Bim, Noxa, Puma, Akt-1* and *TNFRSF1A*) was determined by real-time PCR. (**d**) Liver cells from C57BL/6 mice were isolated and cultured with BALB/c mouse pancreatic islets at a ratio of 10000:1 (1 × 10^5^ liver cells and 10 islets) in the presence or absence of CFSE-labeled JNK1 or control siRNA-transfected Tregs from WT C57BL/6 mice (1 × 10^4^). After 72 hours, the percentages of CFSE+ AnnexinV+ cells were measured by flow cytometry. (**e**) A representative flow cytometry figure. Mean values, p values and SEs are shown. Data presented are representative of three independent experiments.

The above experiment demonstrates that JNK1-deficient Tregs survive better in liver parenchyma of CDM recipient compared to WT Tregs. We asked whether the lack of JNK1 expression makes Tregs less apoptotic. We isolated WT or JNK1^−/−^ Tregs and stimulated them with isotype control antibodies IgG and IgG2 or anti-CD3 and anti-CD28 antibodies as mentioned in the Methods section. As shown in Fig. 3a, after 6 hours, the percentage of AnnexinV+ JNK1^−/−^ Tregs was significantly less than the number of AnnexinV + WT Tregs (0.54 ± 0.30 *vs*. 4.61 ± 1.80, p < 0.01, Fig. 3a). We also determined the expression of various pro- and anti-apoptotic genes in the above-cultured cells by real-time PCR and western blot analysis. As shown in Fig. 3c, expression of the anti-apoptotic genes *Mcl-1* and *Bcl-xL* was significantly higher in activated JNK1^−/−^ Tregs than in activated WT Tregs (*Mcl-1* 0.7 ± 0.18 *vs*. 1.5 ± 0.32, p < 0.05 and *Bcl-xL* 0.20 ± 0.18 *vs*. 1.2 ± 0.27, p < 0.001). In contrast, activated WT Tregs expressed significantly higher levels of the pro-apoptotic genes *Bcl-2-like protein 11* (*Bim)* (3.3 ± 0.18 *vs*. 2.2 ± 0.27, p < 0.05)*, p53 upregulated modulator of apoptosis (puma)* (2.4 ± 0.23 *vs*. 1.4 ± 0.02, p < 0.01) and *tumor necrosis factor receptor superfamily member 10B (TNFSRSF10B)* (2.4 ± 0.23 vs. 1.4 ± 0.02 p < 0.01) compared with activated JNK1^−/−^ Tregs (Fig. 3c). We also determined bcl-2-like protein 4 (Bax), Mcl-1, Bim, Phorbol-12-myristate-13-acetate-induced protein 1 (Noxa), and B-cell lymphoma 2 (bcl-2) protein expression by western blotting. We isolated Tregs from WT C57BL/6 mice and treated them with control siRNA or JNK1 siRNA stimulated them with anti-CD3 and anti-CD28 antibodies as mentioned in the Methods section. As shown Supplementary Fig. 4, expression of the anti-apoptotic genes *Mcl-1* was higher in JNK1 siRNA treated compared to the control siRNA WT Tregs. In contrast, activated WT Tregs treated with control siRNA expressed higher levels of the pro-apoptotic protein *Bim* compared to JNK1 siRNA treated Tregs. To determine whether JNK1-defective Tregs are less apoptotic under a more physiological condition, we isolated Tregs from WT C57BL/6 mice and treated them with control siRNA or JNK1 siRNA and then labeled the cells with carboxyfluorescein succinimidyl ester (CFSE). JNK1 siRNA inhibited JNK1 mRNA expression by 75–80%, as quantified by real-time PCR (Supplementary Fig. 2a). The siRNA knockdown was effective up to 8 to 10 days in cultured cells. Liver cells from C57BL/6 mice were isolated and cultured with BALB/c mouse pancreatic islets at a ratio of 10000:1 (1 × 10^5^ liver cells and 10 islets) in the presence or absence of JNK1 siRNA-transfected CFSE-labeled Tregs (1 × 10^4^). After 24 hours, the percentage of CFSE+Annexin V+ cells was determined by flow cytometry. As shown in Fig. 3d,e, JNK1 siRNA significantly inhibited apoptosis (fewer AnnexinV+CFSE+ cells). In contrast, control siRNA had no effect on apoptosis in Tregs (Fig. 3d,e).

### JNK1^−/−^ Tregs produce more IL-10 and TGF-β and express more LAG-3 than WT Tregs

**Figure 4:**
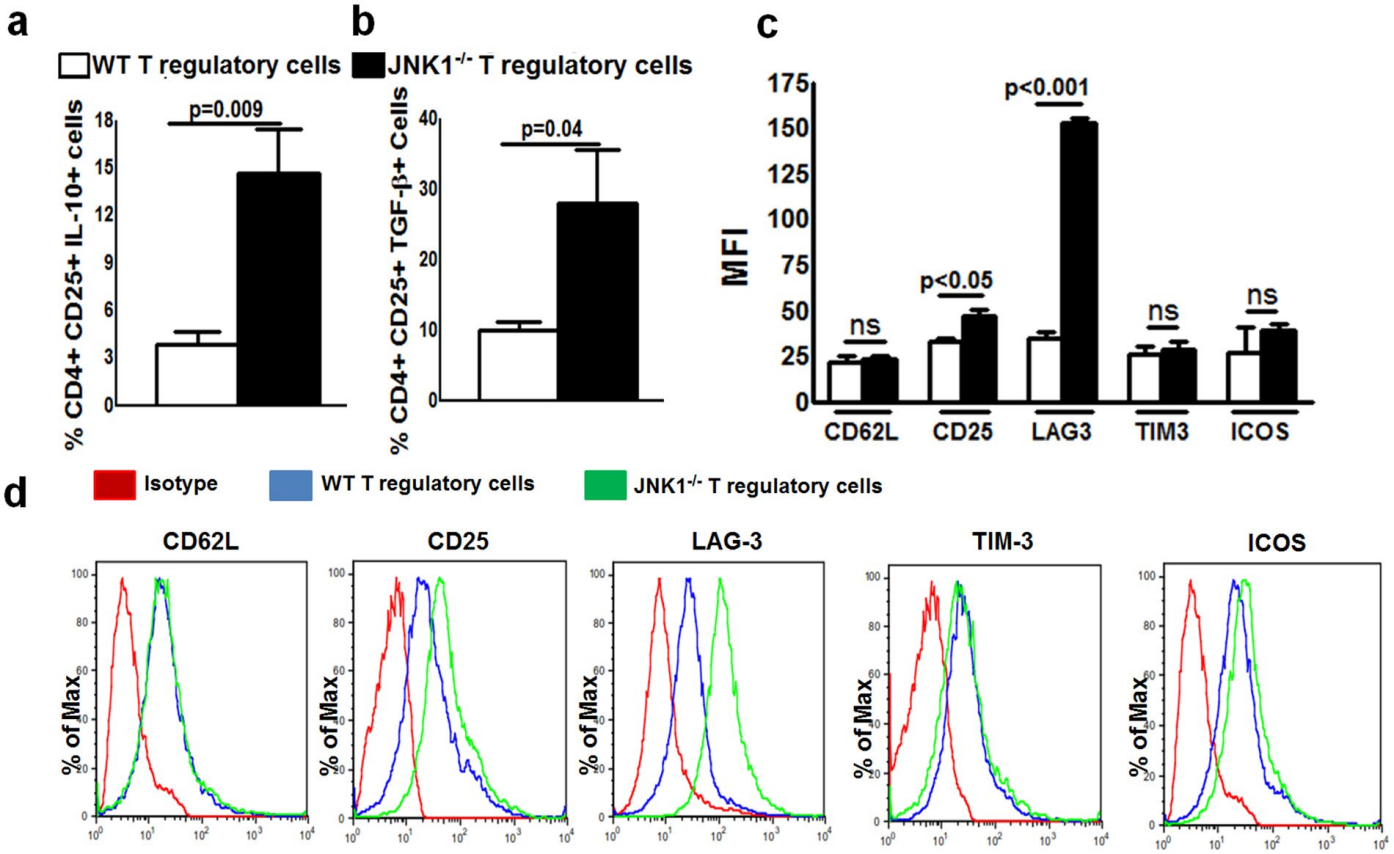
JNK1^−/−^ Tregs produce more IL-10 and TGF-β and express more LAG-3 than WT Tregs. CD4+CD25+ Tregs from WT or JNK1^−/−^ were stimulated with anti-CD3 (5 μg/ml) or anti-CD3 anti-CD28 (1 μg/ml) as described in the Methods section. After 72 hours, the percentage of (**a**) IL-10 and (**b**) TGF-β producing cells was determined. (**c**) Mean fluorescence intensity (MFI) of LAG-3, CD62L, ICOS, CD25 and TIM-3 expression by WT and JNK1^−/−^ Tregs. (**d**) A representative flow cytometry figure. Mean values, p values and SEs are shown. Data presented are representative of three independent experiments.

**Figure 5:**
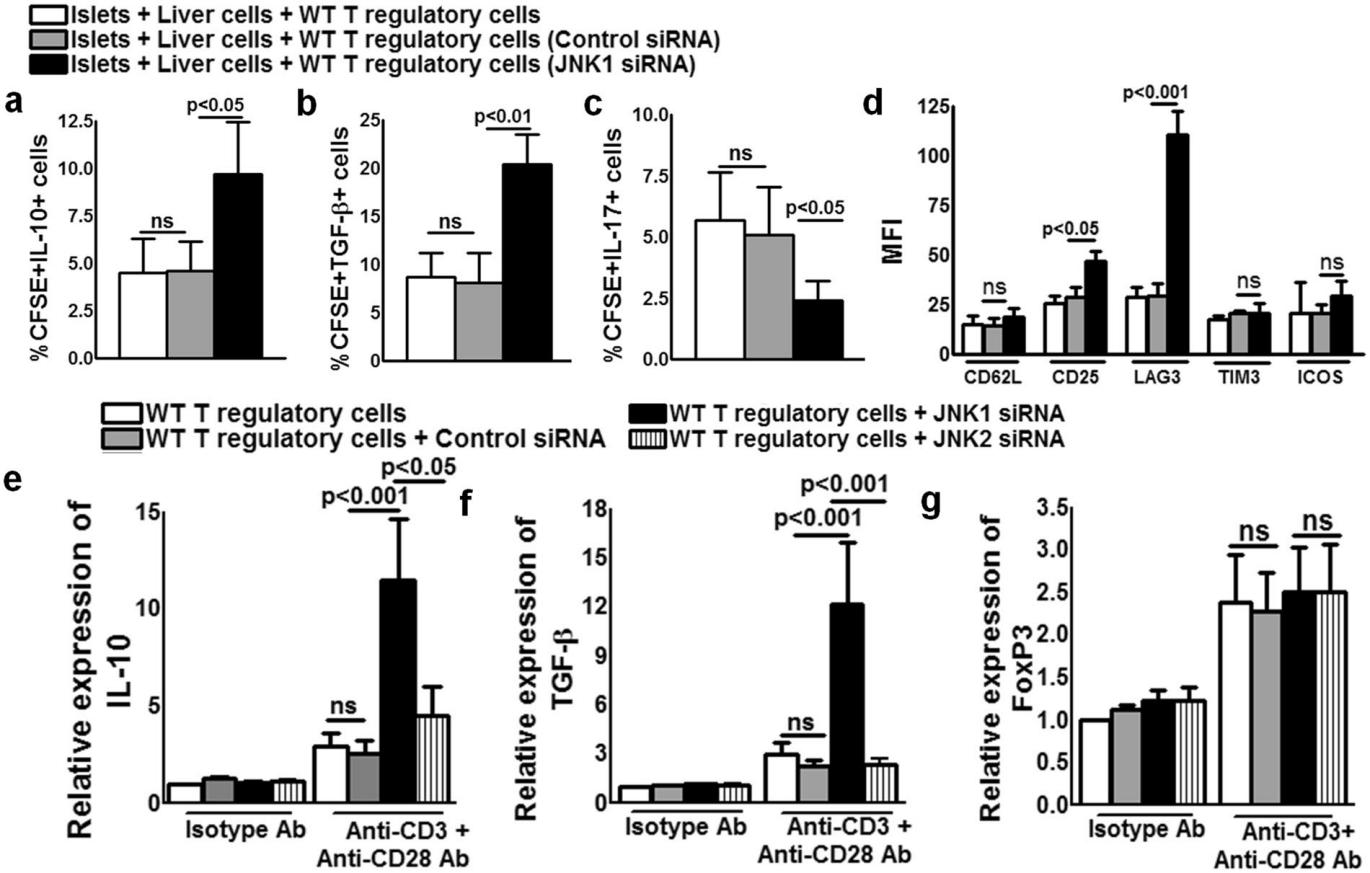
JNK1 siRNA enhances WT Treg production of IL-10 and TGF-β. a to c. Liver cells from C57BL/6 mice were isolated and cultured with BALB/c mouse pancreatic islets at a ratio of 10000:1 (1 × 10^5^ liver cells and 10 islets) in the presence or absence of freshly isolated CFSE-labeled and JNK1 or control siRNA-transfected Tregs from WT C57BL/6 mice (10^4^). After 72 hours, the percentage of Annexin V + cells expressing (**a**) IL-10, (**b**) TGF-β, (**c**) IL-17, (**d**) LAG3, CD25, CD62L, ICOS and TIM-3 was measured by flow cytometry. Tregs from WT C57BL/6 mice were isolated and transfected with JNK1 or JNK2 or control siRNA as described in the Methods section. Twelve hours after transfection, the cells were stimulated with anti-CD3 (5 μg/ml) or anti-CD3 and anti-CD28 (1 μg/ml). After 72 hours, real-time PCR analysis was performed to evaluate gene expression. (**e**) IL-10 (**f**) TGF-β and (**g**) Foxp3. Mean values, p values and SEs are shown. Data presented are representative of three independent experiments.

In the above cultured cells, intracellular staining indicated that JNK1^−/−^ Tregs or JNK1 siRNA-transfected cells produced more IL-10, TGF-β and less IL-17 than WT type Tregs or control siRNA-transfected cells (Figs 4a,b and 5a–c). We also determined the expression of Treg surface markers. As shown in Figs 4c–e and 5d, the lack of JNK1 expression significantly enhanced the surface expression of LAG-3 in comparison to WT Tregs. Baseline expression of LAG-3 by JNK1^−/−^ Tregs was elevated, and it was further increased by culturing the cells with cross-linked anti-CD3 and anti-CD28 antibodies (Supplementary Fig. 2b). WT Tregs treated with JNK1 siRNA significantly enhanced LAG-3 expression and resulted in fewer apoptotic cells in comparison to control siRNA-treated cells (Supplementary Fig. 5). Expression of CD25 was marginally enhanced in JNK1^−/−^ Tregs compared to WT Tregs. There is no difference in the expression of CD62, ICOS and TIM-3 between JNK1^−/−^ Tregs and WT Tregs or CFSE+ control siRNA or JNK1 siRNA transfected cells (Figs 4c,d and 5d).

As an additional means of determining whether the lack of JNK1 enhanced IL-10 and TGF-β expression in response to stimulation with anti-CD3 and anti-CD28 antibodies, we isolated CD4+CD25+ Tregs from WT C57BL/6 mice and treated them with control siRNA or JNK1 siRNA or JNK2 siRNA (as an additional control), followed by stimulation with anti-CD3 and anti-CD28 antibodies. As shown in Fig. 5e,f, JNK1 siRNA significantly enhanced IL-10 and TGF-β gene expression. In contrast, JNK1 siRNA had no effect on Foxp3 gene expression (Fig. 5g) and JNK2 siRNA had no effect on Foxp3, IL-10 and TGF-β gene expression (Fig. 5e–g).

### JNK1^−/^ but not WT Tregs inhibit IL-17 and IL-21-mediated inflammatory responses

**Figure 6:**
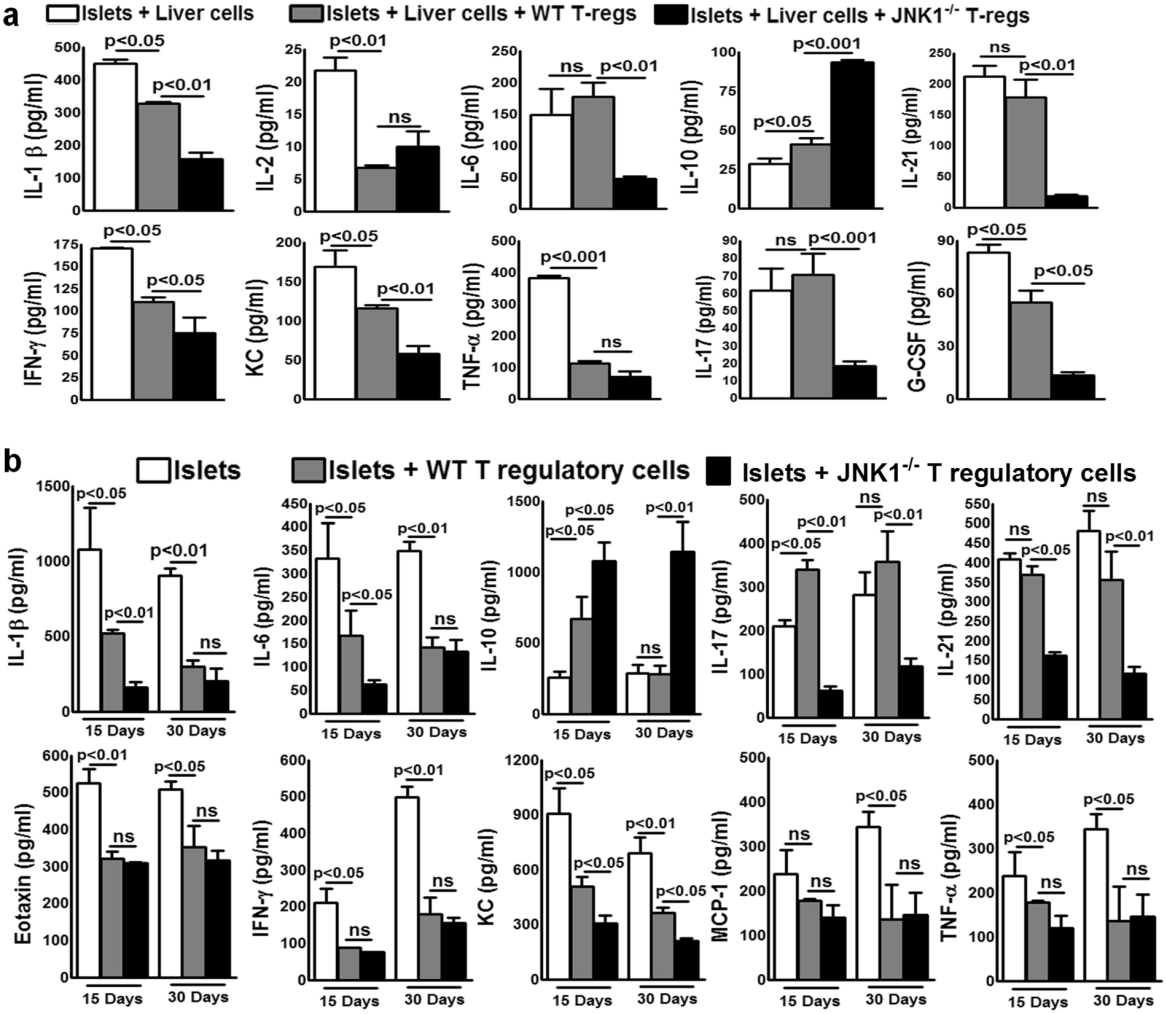
JNK1^−/−^ but not WT Tregs inhibit IL-17 and IL-21-mediated inflammatory responses (**a**) Liver cells from C57BL/6 mice were isolated and cultured with BALB/c mouse pancreatic islets at a ratio of 10000:1 (1 × 10^5^ liver cells and 10 islets) in the presence or absence of Tregs from WT C57BL/6 or JNK1^−/−^ (1 × 10^4^) mice. After 72 hours, various cytokine levels were measured by multiplex ELISA. Data from three independent experiments are shown. Mean values, p values and SEs are shown (**b)**. Pancreatic islets and Tregs from WT or JNK^−/−^ mice were transferred to CDM as described in Fig. 1. Fifteen and thirty days after transplantation, liver cell homogenates were prepared, and various cytokine levels were measured by multiplex ELISA. Data from five independent experiments are shown. Five mice per group were used. Mean values, p values and SEs are shown.

We determined the immunosuppressive capacity of WT and JNK1^−/−^ Tregs on C57BL/6 mouse liver cells by measuring the production of pro-and –anti-inflammatory cytokines in the presence of BALB/c mouse pancreatic islets. Liver cells from C57BL/6 mice were isolated and cultured with BALB/c mouse pancreatic islets at a ratio of 10000:1 (1 × 10^5^ liver cells and 10 islets) in the presence or absence of Tregs from WT C57BL/6 or JNK1^−/−^ (1 × 10^4^) mice. After 72 hours, various cytokine levels were measured by multiplex ELISA. As shown in Fig. 6a, WT and JNK1^−/−^ Tregs showed reduced production of IL-1β, IL-2, G-CSF, IFN-γ, TNF-α and KC in comparison to liver cells and islets cultured without Tregs. JNK1^−/−^ Tregs inhibited the production of the above cytokines more efficiently (Fig. 6a). In addition, JNK1^−/−^ Tregs inhibited the production of IL-6, IL-17 and IL-21 by liver cells (Fig. 6a). In contrast, WT Tregs were unable to inhibit the production of these cytokines.

In Fig. 1, we show that the median graft survival time was 18 days in the liver parenchyma of mice transplanted with only islets, 40 days in mice that received islets and WT Tregs and 120 days in mice that received islets and Tregs from JNK1^−/−^ mice. To evaluate the *in vivo* relevance of the above findings, pancreatic islets from BALB/c mice were transplanted into the liver parenchyma of C57BL/6 CDM with or without WT or JNK1^−/−^ Tregs, as described in the Methods section. Fifteen and 30 days post-islet transplant, liver homogenates were prepared, and cytokine and chemokine levels were determined by multiplex ELISA. As shown in Fig. 6b, after 15 days, WT and JNK1^−/−^ Tregs inhibited the production of IL-1β, IL-6, eotaxin, IFN-γ, KC and TNF-α. In contrast, WT Tregs were unable to inhibit IL-17 and IL-21 cytokine production. Thirty days after transplantation, JNK1^−/−^ Tregs inhibited the production of IL-6, IL-17 and IL-21 and enhanced IL-10 production compared with WT Tregs. One hundred twenty days after transplantation, JNK1^−/−^ Tregs were unable to inhibit the production of IL-17 and IL-21 and showed enhanced production of IL-1β, TNF-α, IL-6 and IFN-γ after 15 days when islets alone and 30 days when islets together with WT Tregs were transplanted into mice (Supplementary Fig. 3a).

### LAG-3 and IL-10 but not TGF-β are responsible for JNK1^−/−^ Treg-mediated inhibition of IL-21 and IL-17 production

**Figure 7:**
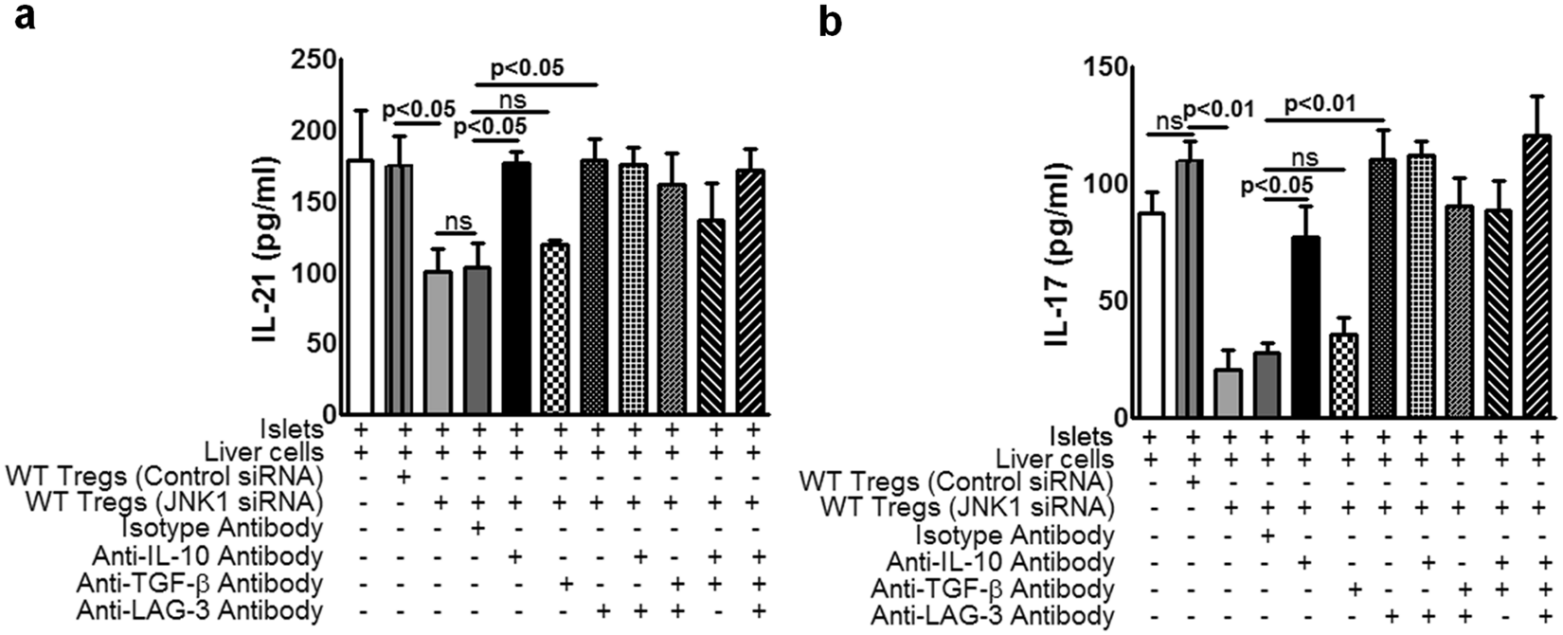
LAG-3 and IL-10 but not TGF-β are responsible for JNK1^−/−^ Treg-mediated inhibition of IL-21 and IL-17 production. Liver cells from C57BL/6 mice were isolated and cultured with BALB/c mice pancreatic islets at a ratio of 10000:1 (1 × 10^5^ liver cells and 10 islets) in the presence or absence of WT Tregs treated with control or JNK1 siRNA and antibodies against LAG-3 or IL-10 or TGF-β or isotype controls (10 µg/ml). In some wells, a combination of LAG-3, IL-10, TGF-β antibodies (suboptimal dose 2. 5 µg/ml each) was used. After 72 hours, (**a**) IL-21 and (**b**) IL-17 levels were measured by multiplex ELISA. Data from three independent experiments are shown. Mean values, p values and SEs are shown.

In Fig. 3, we show enhanced production of IL-10 and TGF-β and increased expression of LAG-3 by activated JNK1^−/−^ Tregs. We next asked whether the JNK1^−/−^ Treg-mediated inhibition of IL-17 and IL-21 was dependent on these molecules. Liver cells from C57BL/6 mice were isolated and cultured with BALB/c mouse pancreatic islets at a ratio of 10000:1 (1 × 10^5^ liver cells and 10 islets) in the presence or absence of WT Tregs treated with control or JNK1 siRNA (1 × 10^4^). Some of the JNK1siRNA-treated Tregs were cultured together with islets and liver cells in the presence of anti-LAG-3, anti-IL-10, anti-TGF-β, or isotype control antibodies. After five days, IL-21 and IL-17 levels were measured by ELISA. Anti-LAG-3 or anti-IL-10 antibodies significantly increased the production of IL-17 and IL-21 (Fig. 7). In contrast, anti-TGF-β or isotype control antibodies had no effect on IL-17 and IL-21 production (Fig. 7). As shown in Fig. 7, addition of antibodies to LAG-3 and IL-10 together to the above cultured cells increased IL-17 and IL-21 production similarly to individual antibody treatments. LAG-3 siRNA treatment of JNK1^−/−^ Tregs also increased IL-21 production (Supplementary Fig. 3b). Our above findings suggest that JNK1 siRNA-treated WT Tregs inhibit IL-17 and IL-21 production by liver cells through LAG-3 and IL-10 independently.

## Discussion

In the current study, we found that transplantation of allogeneic pancreatic islets along with Tregs defective in JNK1 signaling prolonged islet allograft survival in the liver parenchyma of CDM. We also found that adoptively transferred JNK1^−/−^ Tregs were less apoptotic and persisted for a longer period of time in the transplanted liver compared with WT Tregs. Furthermore, we found that JNK1^−/−^ but not WT Tregs inhibited IL-17 and IL-21-mediated inflammation in the liver parenchyma. JNK1^−/−^ Tregs expressed significantly higher levels of LAG-3 and produced more IL-10 to inhibit IL-17 and IL-21-mediated inflammation. Our study identifies a novel role of JNK1^−/−^ Tregs to prolong allograft survival in liver parenchyma.

Tregs are a specialized subset of T cells that are able to migrate to an inflamed graft site, decrease inflammation, improve graft survival and facilitate self-tolerance^9^. Recent advances in technology have allowed the effective isolation and expansion of Tregs *ex vivo* and adoptive transfer to control allo- and autoimmune responses^26–28^. In allogeneic islet transplantation, localized inhibition of inflammation and induction of tolerance is essential because systemic application of Tregs requires very high numbers of cells to achieve optimum efficacy^28–30^. Furthermore, because the systemically administered Tregs require chemokine trafficking to find the graft site^31^, they can develop immunologic unresponsiveness and increased susceptibility to infections and cancer^32,33^. Pancreatic islet transplantation to immune-privileged sites such as the kidney capsule, testis, liver and encapsulated islets in the bloodstream can protect islets from inflammation^34–36^. Among various immune-privileged sites, pancreatic allograft can survive long term in liver parenchyma^21,22^. In the current study, we loaded allogeneic BALB/c mouse pancreatic islets with Tregs from C57BL/6 mice and transplanted them into the liver parenchyma of C57BL/6 CDM. We found that islets pre-populated with Tregs survived for a longer period of time (40 days) than islets alone (18 days) in the liver parenchyma of CDM (Fig. 1b). We also found that Treg-pre-populated islets survive longer in the liver parenchyma of NOD mice (Supplementary Fig. 1c–e). Allogeneic pancreatic islet survival was further increased to 120 days (Fig. 1b) when the islets were loaded with JNK1^−/−^ Tregs.

JNK1 belongs to the mitogen–activated protein kinase (MAPK) family consisting of four members: extracellular-signal-regulated-kinases (ERKs), ERK5 (MAPK7), JUN N-terminal (JNKs) and P38 kinase^37^. MAPKs play an important role in T cell growth, differentiation and apoptosis^17,38^. P38 MAPK activation is required for the maintenance of T cell anergy, while ERK and JNK MAPK signal transduction is defective in anergic CD4+ T cells^39–41^. The role of MAPK pathways in Tregs remains unclear^42–46^. In Tregs, P38 MAPK activity is markedly enhanced, the activation of the JNK pathway is significantly reduced and Ras-ERK activity is defective^47,48^. It is unknown whether activation or deactivation of these MAPKs is essential for the suppressor function of naturally occurring Tregs^49^. JNK1 inhibits Th2 responses during *Leishmania major* infections^50^. The absence of JNK1 in T cells represses recall proliferative responses^18^. Splenocytes from JNK1^−/−^ mice secrete lower amounts of Th1 cell-associated cytokines and chemokines in response to TCR stimulation^18,51^. The absence of JNK1 promotes IL-4 and IL-10 production by T cells^18^. In the current study, we found that JNK1-defective Tregs survived longer, expressed higher levels of LAG-3 molecule, produced higher levels of IL-10 and TGF-β, were more immunosuppressive and prolonged allograft survival in comparison to WT Tregs.

Tregs proliferate more rapidly than conventional T cells under static conditions^52^. We found adoptively transferred JNK1^−/−^ Tregs persisted (Fig. 2b) and survived longer than WT Tregs in liver parenchyma (Fig. 2b). TCR-dependent proliferation of Tregs is regulated by mTOR signaling pathways^53^ and transcription factors such as STAT5 and NF-κB activation^13,54^. Our studies further demonstrated that the lack of JNK1 enhanced the proliferative capacity of Tregs in an inflammatory environment. JNK1 activation regulates the activity of pro- and anti-apoptotic transcription factors^15,23^. JNK1 functions as a proapoptotic kinase^15^, and cells from JNK1^−/−^ mice become resistant to apoptosis^37^. We confirmed the findings that JNK1^−/−^ Tregs are less apoptotic (Fig. 3c) and, upon TCR stimulation, express higher levels of the anti-apoptotic genes *Mcl-1* and *Bcl-XL* and lower levels of the pro-apoptotic genes *TNFRSF10B, Bim* and *Puma* compared with WT Tregs. We further found that the lack of JNK1 expression enhanced the capacity to express LAG-3 and produce IL-10. Excess IL-10 is known to inhibit apoptosis^55^, and our current findings demonstrated that JNK1^−/−^ Tregs were less apoptotic and provided enhanced tolerance to allografts more efficiently than WT Tregs.

Treg-mediated suppression represents a unique, cell extrinsic mechanism that negatively regulates immune cells and inhibits inflammation^56^. We found that JNK1^−/−^ Tregs inhibited IL-1β, IL-2, IL-6, eotaxin, IFN-γ, TNF-α and KC production and enhanced IL-10 production in liver parenchyma more efficiently than WT Tregs. In addition, JNK1^−/−^ but not WT Tregs inhibited IL-17 and IL-21 production. Activation of Tregs in the presence of antigen-presenting cells (APC) induces the differentiation of Tregs into IL-17 producers, and IL-1β is mandatory for this function^57,58^. JNK1^−/−^ lymphoid cells are profoundly defective in IL-17 production compared with WT cells^18^. We found that culturing isolated JNK1^−/−^ Tregs, islets and liver cells together inhibited IL-17 and IL-21 production in comparison to co-culturing islets and liver cells. In contrast, WT Tregs had no effect on IL-17 and IL-21 production. JNK1 siRNA significantly reduced the capacity of WT Tregs to produce IL-17 and IL-21 (Fig. 5c). IL-17 is a pleiotropic cytokine with multiple pro-inflammatory functions and is involved in allograft rejection. IL-17 up-regulates neutrophil chemoattractant molecules and enhances early neutrophil influx into allografts, thus facilitating further recruitment of pathogenic IFN-γ-producing T cells into the graft^59,60^. IL-21 is another Th17 cytokine produced by activated CD4+ T cells and NK cells that directly contributes to the orchestration of the different pathways that regulate the immune response^61^. IL-21 has a role in graft versus-host disease^62^ and is an important player in the alloimmune response; it robustly expands effector T cells and inhibits TGF-β driven differentiation of naïve T cells into Tregs and inhibits Treg effector functions^63^. IL-21 is an anti-FoxP3/anti-Treg cytokine that functions in the late-phase alloimmune response to disrupts FoxP3 and alter Treg functions, thereby acting as an antitolerogenic factor^61^. Thus, IL-21 elicits an enhanced alloimmune response and creates a barrier to transplant tolerance^61^. Our results suggest that the lack of JNK1 signaling in Tregs inhibits their capacity to produce IL-17 and inhibits IL-17 and IL-21 production in an inflammatory environment.

We found that activated JNK1^−/−^ Tregs expressed higher levels of LAG-3 and produced more IL-10 and TGF-β compared with WT Tregs. We also found that IL-10 and LAG3, but not TGF-β, inhibited IL-17 and IL-21 production. LAG-3 is a CD4-related molecule that is highly expressed on activated Tregs^64^. Binding of LAG-3 to MHC class II molecules induces immunoreceptor tyrosine-based activation motif (ITAM)-mediated inhibitory signaling^65^. Anti-LAG-3 antibody completely blocks the Treg-mediated protection from lethal pulmonary vasculitis^64^. LAG-3+ Tregs are more immunosuppressive and produce more IL-10 than LAG-3- Tregs^64^. LAG-3+ cells suppress colitis in an IL-10-dependent manner^66,67^. IL-10 negatively regulates the expression of IL-17 and *RORγt* by both macrophages and T cells^68^. We found that LAG-3 and IL-10 independently (no synergic effect) inhibited IL-17 and IL-21 production (Fig. 7a,b). Our findings demonstrate that defective JNK1 signaling leads to increased LAG-3 expression and enhanced IL-10 production by Tregs which inhibits IL-17 and IL-21 production.

In summary, we found that JNK1^−/−^ Tregs inhibit inflammatory immune responses to islet allografts and prolong allograft survival in liver parenchyma more efficiently than WT Tregs. We also found that JNK1^−/−^ Tregs are stable and functional for longer periods of time than WT Tregs in the liver inflammatory environment. Further understanding of the signaling pathways in JNK1^−/−^ Tregs that prevent apoptosis and enhance their immunosuppressive capacity will facilitate the development of immunosuppressive therapies to prevent allograft rejection.

## Methods

### Animals

Six to eight-week-old female wild-type (WT) C57BL/6-(CD45.2), C57BL/6-(CD45.1), JNK1 knockout (JNK1^−/−^) C57BL/6 background, non-obese diabetic (NOD)/ShiLtJ, C3H/HeJ and 11 to 12-week-old female BALB/c mice were purchased from The Jackson Laboratories (Bar Harbor, ME, USA). The Institutional Animal Care and Use Committee of the University of Texas Health Science Center at Tyler approved the studies. All animal procedures involving the care and use of mice were in accordance with the guidelines of NIH / OLAW (Office of Laboratory Animal Welfare).

### Induction of diabetes

Diabetes in WT C57BL/6 mouse was induced by administration of streptozotocin (STZ) (Sigma-Aldrich). STZ was dissolved in a 50 mM citric acid buffer and administered (180 mg/kg of body weight) intraperitoneally one time. STZ solution was stored on ice for less than 30 minutes before injections were performed. Mice were fasted for 16 hours before the STZ injection.

### Blood glucose determination

Prior to diabetes induction, blood glucose levels were measured in the submandibular vein blood using a Bayer Contour blood glucose meter. After STZ injection, blood glucose and animal health were monitored 2 times per week. Chemically induced diabetes (CDM) was diagnosed when blood sugars exceeded 300 mg/dL for 3 consecutive readings, but transplantation was not performed unless blood sugars were substantially higher, preferably in excess of 400 mg/dL. Control mouse blood glucose levels were consistently between 80 and 100 mg/dl. All blood glucose measurements were obtained in the fed state early in the afternoon to eliminate variability in blood glucose levels caused by the feeding patterns of the mice.

### Abs and other reagents

For flow cytometry, we used FITC anti-CD4, APC anti-CD8, FITC anti-IgM, APC anti-CXCR5, PE anti-PD1, FITC anti-CD62L, PE anti-CD25, APC anti-LAG-3, APC anti-TIM-3, PE anti-PD-1 and FITC anti-ICOS, FITC anti-Annexin V and APC anti-FoxP3 antibodies (all from BioLegend).

### Islet isolation

Islets were isolated from BALB/c or C57BL/6 or C3H/HeJ mice by collagenase V and VI digestion (Sigma-Aldrich) of the pancreas and purified using a density gradient method^69^. Briefly, mice were euthanized with carbon dioxide. An incision was made to open the upper abdominal cavity, and 4 ml of collagenase V (2 mg/ml) and VI (0.5 mg/ml) cocktail solution was injected into the common bile duct to inflate the pancreas. After inflation, the pancreas was excised and digested at 37 °C for 5 min. The digested pancreas was loaded on Histopaque (1.10 g/ml, Sigma Aldrich) solution and centrifuged at 600 g for 20 minute to separate the islets. The islet layers collected on the interface of the islet cell clumps were centrifuged by washing twice with Hank’s solution containing 10% fetal bovine serum at 300 g for 2 min. Islet pellets were dissolved, placed in a 100-mm petri dish and hand-picked under a stereomicroscope using a capillary glass tube pipette tip with an inner diameter of 500 μm. After collection, the islet cell clumps were centrifuged by washing once with Hank’s solution containing 10% fetal bovine serum at 300 g for 2 min. The pellet was suspended in RPMI 1640 medium supplemented with 10% FBS and transferred to a 37 °C incubator until use.

### Islet transplantation

NOD or CDM were used as islet recipients. Each individual NOD or CDM was anesthetized by continuous inhalation of isoflurane and oxygen. For mice receiving islets or PBS, a middle abdominal incision of approximately 1 cm was made, and the liver was exposed. The caudate lobe of the liver was exposed with a gentle press. A 22-gauge butterfly needle was used to deliver islets in the caudate lobe. Two hundred islets in 50 μl of pharmaceutical grade PBS (Amresco) or PBS alone were injected into the caudate lobe. The butterfly needle was removed after the injection, which was covered with a cotton tip as quickly as possible. The peritoneal and middle abdominal incisions were closed with ETHILON® Nylon sutures. Some NOD recipient mice were treated with low doses of rapamycin at 0.2 or 0.4 mg/kg daily for 14 days. Mice undergoing islet transplantation were monitored by measuring blood glucose twice daily for two weeks with a Bayer glucometer. Normoglycemia was defined as a blood glucose level <300 mg/dL on two consecutive days.

### Isolation of CD4+CD25+Foxp3+ (Tregs) cells and adoptive transfer

Mice were euthanized with CO_2_, and their spleens were harvested. The cell suspension was collected after filtering the ground spleen through a 200-micron mesh. Following the lysis of erythrocytes, the splenocytes were centrifuged, washed twice with PBS, and counted. The CD4+CD25+ Tregs were purified from splenocytes by autoMACS using the mouse CD4+CD25+ regulatory T cell isolation kit (Miltenyi Biotec., Auburn, CA) according to the manufacturer’s instructions. First, non-CD4 cells were indirectly magnetically labeled. Cells were then labeled with fluorescent PE-CD25 antibody. Non-CD4+ T cells were subsequently depleted over a MACS column. The enriched CD4+ T cells, which had been labeled with PE-CD25, were then incubated with anti-PE micro beads to magnetically label the CD25+ T cells. CD4+CD25+ Tregs were subsequently isolated using the AutoMACS Pro Separator. Cell viability was determined by 0.4% trypan blue staining. Approximately 90 to 95% of isolated CD4+CD25+ cells expressed Foxp3, and <1% of CD4+CD25− cells were Foxp3-, as determined by intracellular staining. Isolated Tregs (10^6^) were cultured with (200) islets in RPMI 1640 medium with 10% FBS for 12 hours in a 37 °C incubator prior to transfer along with islets into CDM liver parenchyma. More than 95% of the cells were viable based on fluorescein diacetate/propidium iodide staining (FDA/PI, Sigma-Aldrich). After incubation, Tregs and islets were washed with pharmaceutical grade PBS, further resuspended in 100 μl of pharmaceutical grade PBS and loaded into a 22-gauge butterfly needle to deliver the islets into the caudate lobe of the liver.

### Islet viability and function

The viability of islets under each condition were analyzed by fluorescein diacetate/propidium iodide staining (FDA/PI, Sigma-Aldrich), which was performed by two independent investigators. To determine islet function, 100 pancreatic islets were cultured in RPMI 1640 medium with 10% FBS and 5.5–10 mM glucose (Sigma-Aldrich). Each incubation step was performed for 120 min at 37 °C in a humidified 5% CO_2_ atmosphere.

### Flow Cytometry

For surface staining, 10^6^ cells were resuspended in 100 μl of staining buffer (PBS containing 2% heat-inactivated FBS) and Abs. Cells were then incubated at 4 °C for 30 min, washed twice, fixed in 1% paraformaldehyde, and assessed using a FACSCalibur flow cytometer (BD Biosciences). In some experiments, intracellular staining was performed according to the manufacturer’s instructions.

### Graft survival analysis

In control and islet transplanted mice, blood glucose levels were measured every 72 hours until termination of the mice. Blood samples were collected by submandibular bleeding using a strip glucometer (Elite, Bayer). Graft survival was calculated as the number of days before diabetes recurrence. The day of diabetes recurrence was defined as the first of 2 consecutive days of random blood glucose >300 mg/dl.

### Liver cell isolation

The control and islet transplanted CDM groups were euthanized and perfused with 10 ml of saline solution (0.5 M EDTA in 1X Hanks Balanced Salt Solution without Ca2+ & Mg2+). The perfused liver was removed and mechanically disintegrated in a petri dish containing 1X PBS. The single cell suspensions of liver cells were filtered through a 70-μm mesh filter, and the resulting cell suspension was centrifuged at 1500 rpm for 5 min.

### Isolation of liver lymphocytes

In some experiments, control group and islet transplanted CDM were sacrificed and single cell suspensions of liver were prepared as described in the liver cell isolation section. Liver lymphocytes were isolated by the differential density gradient method using a Percoll (Sigma Aldrich) density gradient.

### *In vitro* allogeneic co-culture

Whole liver cells (1 × 10^5^) from C57BL/6 CDM were cultured with 10 pancreatic islets from BALB/c mice in 12-well plates with RPMI 1640 containing 10% heat-inactivated fetal bovine serum at 37 °C in a humidified 5% CO_2_ atmosphere for three days. Anti-IL10, anti-TGF-β, anti-PD-1, anti-LAG3 antibody or isotype control antibodies (IgG1 for all molecules) (Bio legend, 1.5 μg/ml) were added to some wells on day “0”. After five days, the culture supernatant and cell lysates were collected and stored at −80 °C for further analysis.

### Treg stimulation assay

CD3e (1 μg/ml, clone 145–2C11, BD Biosciences, isotype IgG) and CD28 (2 μg/ml, clone 37.51, NA/LE, BD Biosciences, isotype IgG2) antibodies were diluted in sterile PBS and incubated in 96-well plates at 4 °C. After an overnight incubation, unbound antibodies were removed by washing 3 times with PBS immediately before cell plating. Freshly isolated CD4+ CD25+Tregs (more than 90% Foxp3+) cells (1.5 × 10^6^ cells/ml in 200 μl of complete RPMI 1640) were plated and incubated at 37 °C in a humidified incubator with 5% CO_2_ until analysis of Treg apoptosis and cell surface markers.

### Preparation of liver cell homogenates for cytokine measurements

Islet recipient mice were sacrificed and their livers rapidly excised, rinsed to remove blood and homogenized in homogenization buffer (PBS containing 0.05% sodium azide, 0.5% Triton X-100, and a protease inhibitor cocktail, pH 7.2, 4 °C). Homogenates were centrifuged at 18,000 rpm for 10 minutes, and the supernatant was used for various experiments. Cytokine levels in the liver homogenate were measured by multiplex ELISA or using individual ELISA kits.

### Multiplex ELISA

In the culture supernatants and liver homogenates, the following 27 cytokines and chemokines were measured using a multiplex ELISA kit (Bio-Rad). The cytokines and chemokines analyzed were IL-1β, IL-1ra, IL-2, IL-4, IL-5, IL-6, IL-7, IL-8 (KC), IL-9, IL-10, IL-12 (p70), IL-13, IL-15, IL-17, basic FGF, eotaxin, G-CSF, GM-CSF, IFN-γ, IP-10, MCP-1 (MCAF), MIP-1α, MIP-1β, PDGF-BB, RANTES, TNF-α, and VEGF.

### Real-time PCR for quantification

RNA was isolated from lymphoid organs and the liver lobes that contained grafts (harvested at various time points after transplantation) using TRIzol (Invitrogen) according to the manufacturer’s instructions. cDNA was generated from 0.5 mg of RNA and random hexamer primers using the Maxima First Strand cDNA Synthesis Kit for RT-qPCR (BIO-RAD) according to manufacturer’s instructions, and real-time PCR was performed. Gene expression of *Bax, Mcl-1, Bcl-2, Bcl-Xl, TNFRSF10B, Bim, Noxa, Puma, Akt-1* and *TNFRSF1A, IL-10, FoxP3 and TGF-β* was quantified using SYBR Green Master Mix (Qiagen) and specific primers (Sigma Aldrich) (Supplemental Table 1) for each of the genes in an ABI Prism 7600. All gene expression levels were normalized to GAPDH and/or β- actin internal controls, and the fold change was calculated using the 2^−ΔΔCT^ method.

### Preparation of CD4+ CD25+ Treg lysates and western blotting

CD4+ CD25+ Tregs were isolated from the spleens of WT mice and stimulated *in vitro* with isotype or anti-CD3 and anti-CD28 antibodies as described above. CD4+ CD25+ Treg lysates were prepared using 1% NP-40 lysis buffer and separated by SDS-PAGE. Next, they were electroblotted onto a polyvinylidene fluoride (PVDF) membrane. After blocking with 5% non-fat dry milk in Tris-buffered saline Tween-20 buffer, the membrane was incubated with primary antibodies at 1:1000 dilutions at 4 C° overnight, followed by anti-rabbit horseradish peroxidase-conjugated secondary antibody at a 1:5000 dilution for 1 hour at room temperature. Protein bands were visualized using the enhanced chemiluminescence detection method.

### siRNA transfection

Freshly isolated Tregs were transfected with JNK1 or LAG-3 or scrambled siRNA (Santa Cruz Biotechnology). Briefly, 10^6^ Tregs were resuspended in 500 µl of transfection medium and transfected with siRNA (6 pmoles). After 6 h, an additional 250 µl of 2X RPMI complete medium was added, and the cells were cultured overnight in a 24-well plate. The next day, the Tregs were washed and used for further experiments. The efficiency of siRNA knockdown was measured by real-time PCR to detect JNK1 or LAG-3 mRNA. The siRNA knockdown was effective up to 8 to 10 days in cultured Tregs.

### Immunofluorescence

Frozen sections of liver (5 μm) were fixed in cold acetone for 10 minutes. After fixation, an FcR blocking step was performed at room temperature for 30 minutes. For insulin staining in the transplanted liver, monoclonal mouse primary anti-insulin (Abcam) and CD45.2 monoclonal mouse FITC-tagged anti-CD45.2 (Rockland Immunochemicals, Inc.) antibodies were used. Subsequently, the slides were washed thoroughly with 1X PBS. Then, the tissue sections were stained with their respective secondary antibodies (goat anti-hamster IgG-Alexa 568). The cells were washed with PBS and mounted with Prolong Gold anti-fading reagent with DAPI (Life Technologies, USA). In some experiments, freshly isolated CD4+CD25+ Tregs (more than 90% Foxp3+) (1.5 × 10^6^ cells/ml in 500 μl of complete RPMI 1640) were plated on poly L-lysine-coated cover slips and stimulated with isotype or anti-CD3 and anti-CD28 antibodies and incubated at 37 °C in a humidified incubator with 5% CO_2_ for six hours, and the cells were stained with anti-LAG3 (cell signaling) and anti-Annexin V (Abcam) and their respective secondary antibodies (donkey anti-rabbit IgG-Alexa 488 and rabbit anti-rat IgG-Alexa-594). The slides were examined and analyzed under a laser-scanning confocal microscope (Zeiss LSM 510 Meta laser-scanning confocal microscope).

### Statistical analysis

Prism 4.03 software (GraphPad Software; San Diego, CA) was used for the statistical analyses. A p value less than 0.05 was considered statistically significant. The results are shown as the mean ± SE. Comparisons between groups were performed by paired, unpaired t tests and one-way ANOVA, as appropriate. Islet allograft survival determined by Kaplan-Meier survival curves was compared between groups by log-rank analyses.

## Electronic supplementary material

Supplementary Information
