# Supplementary material for: c-Jun N-terminal kinase 1 defective CD4+CD25+FoxP3+ cells prolong islet allograft survival in diabetic mice
Source: Sci Rep. 2018 Feb 19;8:3310. doi: 10.1038/s41598-018-21477-9 (PMC5818514; doi:10.1038/s41598-018-21477-9)
Supplement: Supplementary file 1 — Supplementary Information [file 41598_2018_21477_MOESM1_ESM.docx]

**c-Jun N-terminal kinase 1 defective CD4+CD25+FoxP3+ cells prolong islet allograft survival in diabetic mice**

*†Deepak Tripathi,* #Satyanarayana S. Cheekatla,*Padmaja Paidipally, *Radhakrishnan, Rajesh Kumar, *Elwyn Welch, *Ramya Sivangala Thandi, *Amy R. Tvinnereim,*Ramakrishna Vankayalapati

*Department of Pulmonary Immunology, Center for Biomedical Research, University of Texas Health Science Center at Tyler, Tyler, Texas, 75708, USA.

#Current Address: Department of Biotechnology, Gandhi Institute of Technology and Management (GITAM) Institute of Science, GITAM University, Visakhapatnam, Andhra Pradesh, 530045, India.

**Corresponding author:**

†Deepak Tripathi, Pulmonary Immunology, University of Texas Health Science Center, 11937 US Highway 271, Tyler, TX 75708-3154, Telephone (903) 877-7662, fax (903) 877-7989, E-mail: [Deepak.Tripathi@uthct.edu](mailto:Deepak.Tripathi@uthct.edu)

**
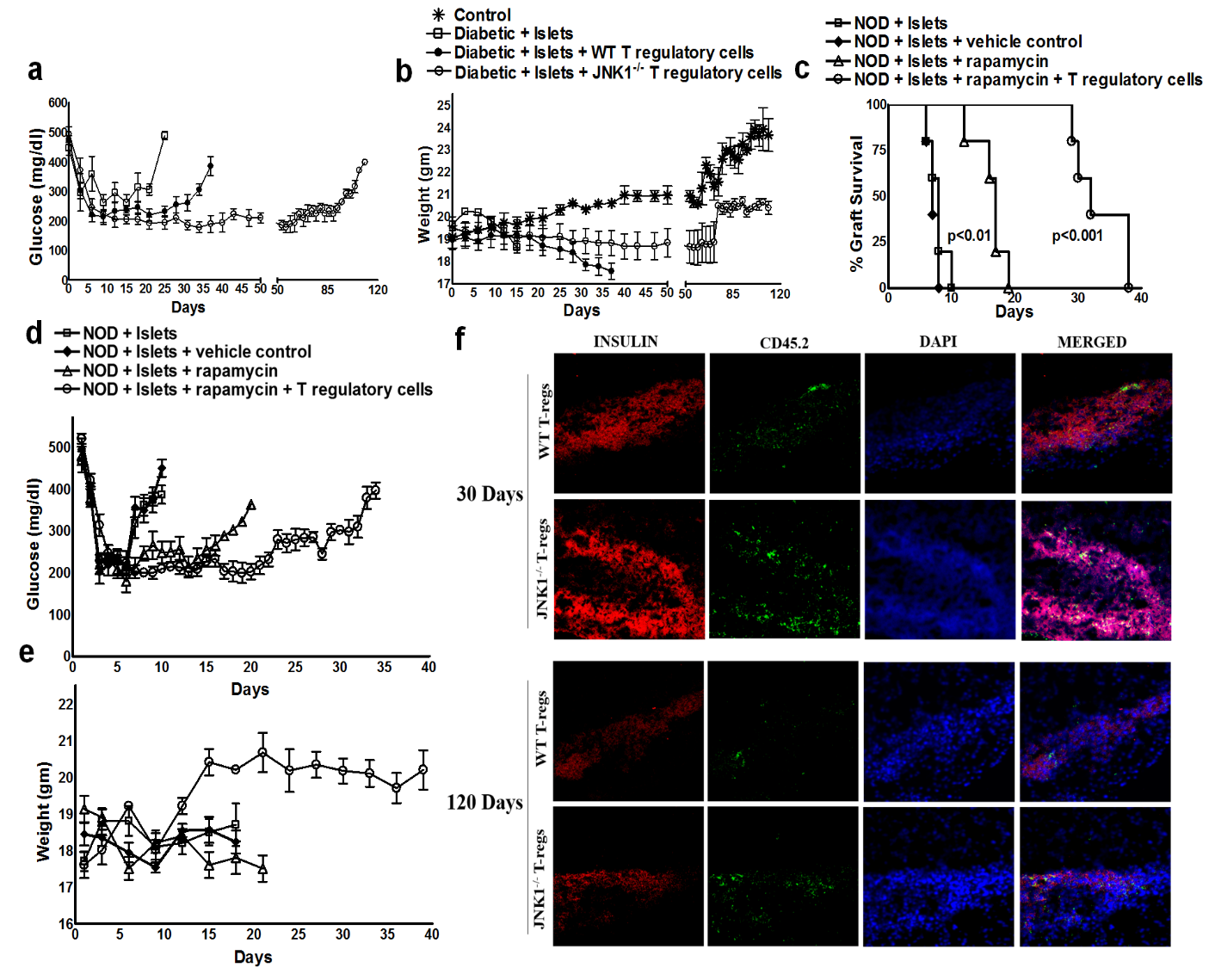
 Supplementary Fig. 1. JNK1 defective Tregs prolong islet allograft survival in liver parenchyma of CDM.** A single intraperitoneal injection of streptozotocin (STZ) (180 mg/kg body weight) caused diabetes in C57BL/6 mice as measured by random blood sugar levels after one week. Approximately 200 pancreatic islets obtained from BALB/c mice (donor) were cultured in medium for 12 hours and transplanted into the liver parenchyma of C57BL/6 CDM (recipient). Some of the islet allograft recipient mice received CD4+CD25+Foxp3+ cells (106) from WT or JNK1^-/-^ mice (both C57BL/6 background) isolated and cultured with islets for 12 hours prior to transfer along with islets as mentioned in the Methods section. (**a)** Blood glucose levels. (**b)** Weight was measured every 72 hours up to 140 days. **(c)** Approximately 200 pancreatic islets obtained from C3H/HeJ mice (donor) were cultured in medium for 12 hours and transplanted into the liver parenchyma of hyperglycemic thirteen-week-old NOD mice (recipient). Some of the islet allograft NOD recipient mice received CD4+CD25+Foxp3+ cells (10^6^) from eight-week-old NOD mice that were isolated and cultured with islets for 12 hours prior to transfer along with islets as mentioned in the Methods section. The percent graft survival was calculated using the log-rank test. Kaplan–Meier survival curves of the mice are shown **(d)** Blood glucose levels. **(e)** Weight was measured every 72 hours up to 40 days. **(f)** Pancreatic islets and Tregs from WT or JNK1**^-/-^** mice (CD45.2) were transferred into CDM mice (CD45.1) as in panel **a**. Thirty and one hundred twenty days after transplantation, the pancreatic islets and co-transplanted CD4+CD25+Foxp3+ (CD45.2) cells in the recipient mouse liver parenchyma were stained with anti-insulin and anti-CD45.2 antibody, respectively, and immunofluorescence images are shown. Data presented are representative of five independent experiments. Five mice per group were assessed.


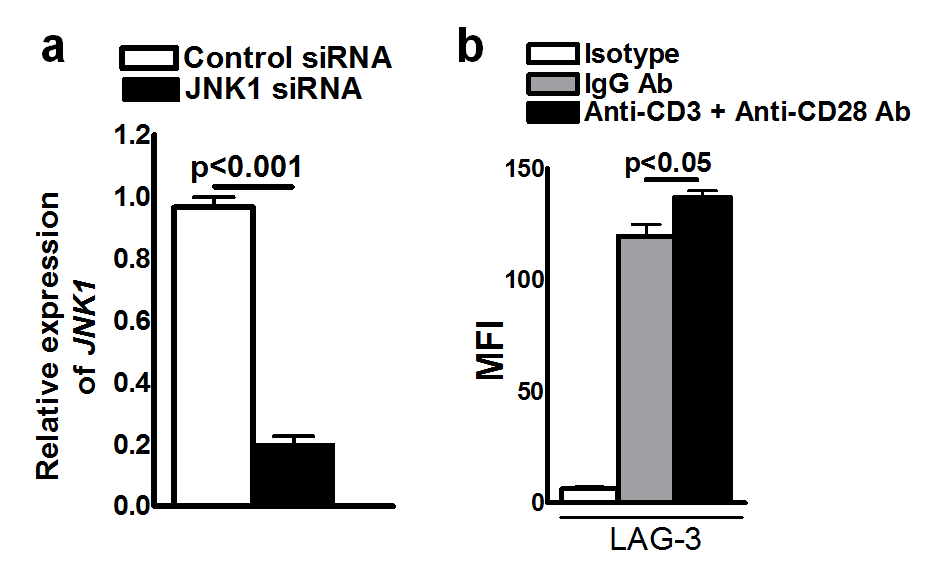
 **Supplementary Fig. 2. (a). Inhibition of JNK1 expression by treatment with JNK1 siRNA.** Tregs (10^4^) from WT C57BL/6 mice were treated with JNK1 or control siRNA, and the level of JNK1 mRNA expression was measured after 72 hours by qRT-PCR. **(b). Baseline expression of LAG-3 in JNK1^-/-^ Tregs.** CD4+CD25+ Tregs from JNK1^-/-^ were stimulated with control anti-CD3 and anti-CD28 antibodies (1 μg/ml) as described in the Methods section. After 72 hours, the mean fluorescence intensity (MFI) of LAG-3 was measured in stimulated and unstimulated JNK1^-/-^ Tregs. Mean values, p values and SEs are shown. Data presented are representative of three independent experiments.

**
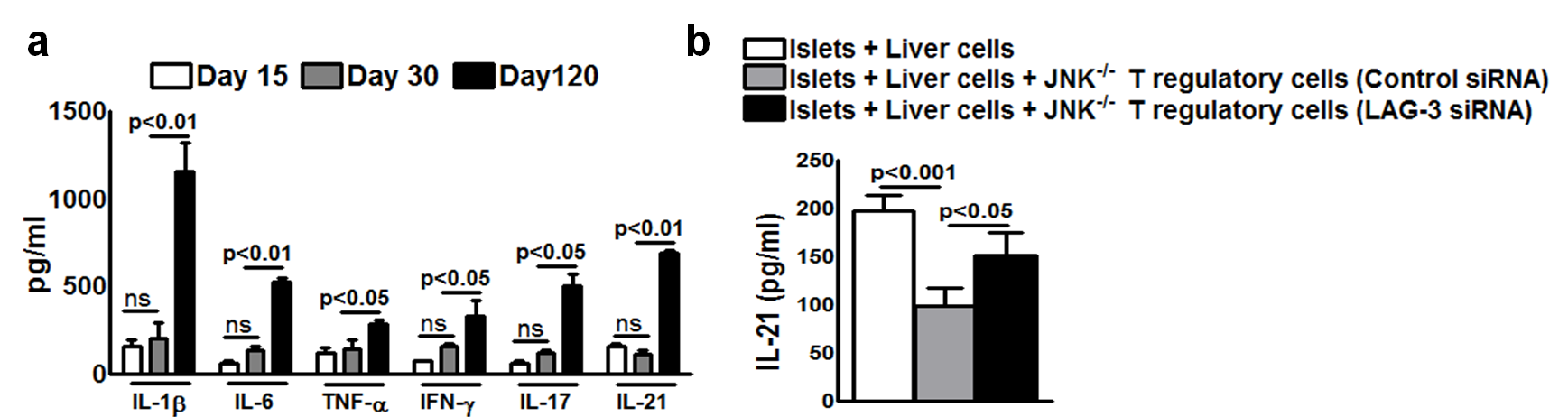
**

**Supplementary Fig. 3. (a) Cytokine levels in the liver parenchyma of islets and JNK1^-/-^ Treg-transplanted CDM.** Pancreatic islets and Tregs from JNK1**^-/-^** mice were transferred to CDM as described in Figure 1. Fifteen, 30, and 120 days after transplantation, liver cell homogenates were prepared, and various cytokine levels were measured by multiplex ELISA. Data from five independent experiments are shown. Five mice per group were used. Mean values, p values and SEs are shown. (**b). LAG-3 expression in JNK1^-/-^ Tregs control IL-21 production**. Liver cells from CDM were isolated and cultured with BALB/c mouse pancreatic islets at a ratio of 10000:1 (10^5^: 10). JNK1^-/-^ Tregs transfected with LAG-3 or scrambled siRNA (control siRNA) were added to some wells of the allogeneic culture. After 72 hours, IL-21 levels were measured by ELISA. Mean values, p values and SEs are shown. Data shown are a representative of three independent experiments.

**
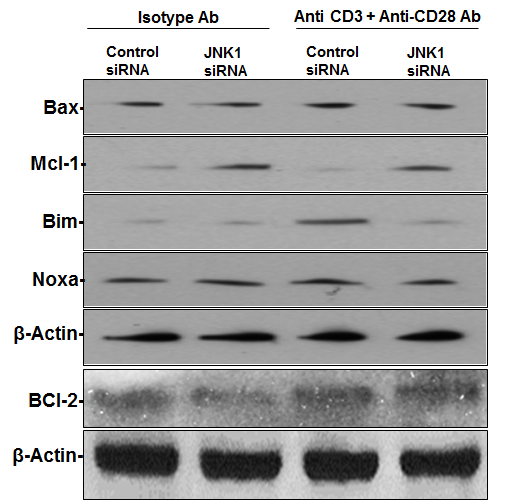
**

**Supplementary Fig. 4. JNK1 siRNA-treated WT Tregs are less apoptotic.** CD4+CD25+ Tregs from WT mice treated with control or JNK1 siRNA and stimulated with isotype control (IgG and IgG2) or anti-CD3 (5 μg/ml) and anti-CD28 (1 μg/ml) antibodies as described in the Methods section. After six hours, the protein expression levels of pro, anti-apoptotic (*Bax, Mcl-1, Bcl-2,* and *Bcl-Xl*) and LAG-3 molecules were determined by western blot analysis. Data presented are representative of three independent experiments.

**
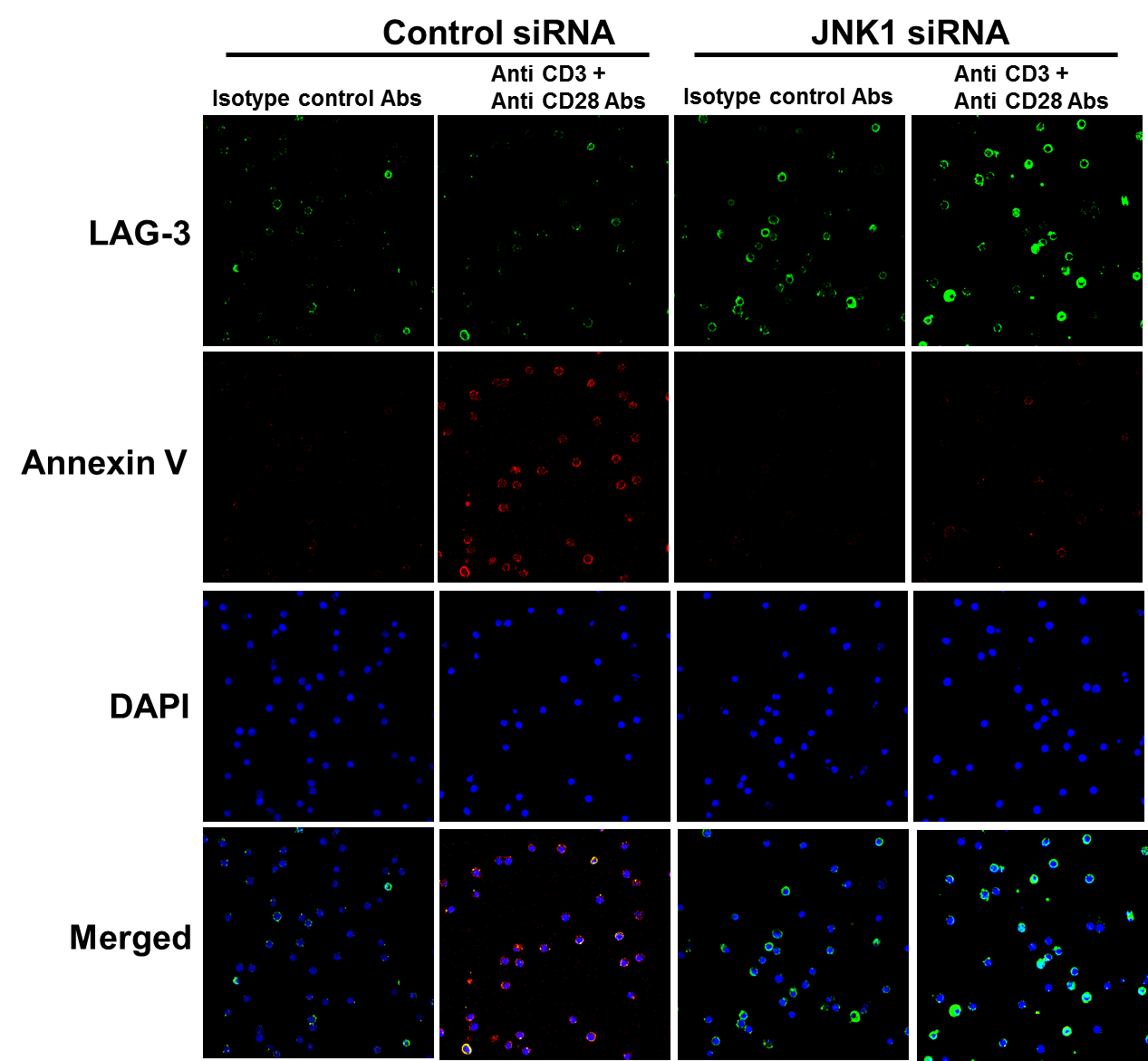
**

**‘**

**Supplementary Fig. 5. JNK1 siRNA enhances WT Tregs to express more LAG-3**

CD4+CD25+ Tregs from WT treated with control siRNA or JNK1 siRNA and stimulated with isotype control (IgG and IgG2) or anti-CD3 (5μg/ml) anti-CD28 (1μg/ml) as described in the methods section. After 6 hours the expression of LAG3 and Annexin V on Tregs was determined by the confocal microscopy. Data presented are representative of three independent experiments.

**Supplemental Table I: List of primers used in this study**

| **S.No** | **Gene name** | **Mouse Primer Sequences** |
| --- | --- | --- |
| 1. | Bcl-2 | Forward: CTGGCATCTTCTCCTTCCAG  Reverse: GACGGTAGCGACGAGAGAAG |
| 2. | Mcl-1 | Forward: TAACAAACTGGGGCAGGATT  Reverse: GTCCCGTTTCGTCCTTACAA |
| 3. | Bcl-xl | Forward: TTCGGGATGGAGTAAACTGG  Reverse: TGGATCCAAGGCTCTAGGTG |
| 4. | Bim | Forward: GAGATACGGATTGCACAGGA  Reverse: TCAGCCTCGCGGTAATCATT |
| 5. | Noxa | Forward: CCCACTCCTGGGAAAGTACA  Reverse: AATCCCTTCAGCCCTTGATT |
| 6. | Puma | Forward: CAAGAAGAGCAGCATCGACA  Reverse: TAGTTGGGCTCCATTTCTGG |
| 7. | TNFRSF10B | Forward: AAGACCCTTGTGCTCGTTGT  Reverse: AGGTGGACACAATCCCTCTG |
| 8. | Akt-1 | Forward: GCACAAACGAGGGGAGTACAT  Reverse: CCTCACGTTGGTCCACATC |
| 9. | TNFRSF1A | Forward: TGCCTACCCCAGATTGAGAA  Reverse: ATTTCCCACAAACAATGGAGTAG |
| 10. | BAX | Forward: GCCCTTTTGCTTCAGGGTTT  Reverse: TCCAATGTCCAGCCCATG |
| 11. | FoXP3 | Forward: CCTGGTTGTGAGAAGGTCTTCG  Reverse: TGCTCCAGAGACTGCACCACT T |
| 12. | IFN-γ | Forward: TCAAGTGGCATAGATGTGGAAGAA  Reverse: TGGCTCTGCAGGATTTTCCATG |
| 13. | TNF-α | Forward: CATCTTCTCAAAATTCGAGTGACAA  Reverse: TGG GAGTAGACAAGGTACAACCC |
| 14. | IL-17 | Forward: CTCCATAAGGCCCTCAGACTAC  Reverse: AGCTTTCCCTCCGCATTGACACAG |
| 15. | IL-1β | Forward: CAACCAACAAGTGATATTCTCCATG  Reverse: GAT CCACACTCTCCAGCT |
| 16. | TGF-β | Forward: GGATACCAACTATTGCTTCAGCTC C  Reverse: AGGCTCCAATATTAGGGGCAGGGT C |
| 17. | IL-10 | Forward: GGTTGCCAAGCCTTATCGGA  Reverse: ACCTGCTCCACTGCCTTGCT |
| 18. | IL-21 | Forward: GCCTCCTGATTAGACTTCGTCAC  Reverse: CAGGCAAAAGCTGCATGCTCAC |
| 19. | JNK1 | Forward: CCACCAAAGATCCCGGACAAG  Reverse: TGGATGCTGAGAGCCATTG |
| 20. | LAG-3 | Forward: AGCTCAATGCCACTGTCACG  Reverse: CAGGTAACCCGAAGGATTTGG |
| 21. | β-Actin | Forward: CTCTGGCTCCTAGCACCATGAAGA  Reverse: GTAAAAGACAGCTCAGTAACAGTCCG |
